# Newer generation straight humeral nails allow faster bone healing and better functional outcome at mid-term

**DOI:** 10.1186/s13018-021-02776-w

**Published:** 2021-10-20

**Authors:** Fabrizio Mocini, Giuseppe Rovere, Domenico De Mauro, Edoardo Giovannetti De Sanctis, Amarildo Smakaj, Giulio Maccauro, Francesco Liuzza

**Affiliations:** Orthopedic Institute, Fondazione Policlinico Universitario A. Gemelli IRCCS - Università Cattolica del Sacro Cuore, Largo Agostino Gemelli n°8, Rome, Italy

**Keywords:** Humeral fractures, Humeral nailing, Fixation, Nail design

## Abstract

**Purpose:**

Although proximal and diaphyseal humerus fractures are frequent, the optimal management remains controversial. Antegrade nailing prevents further damage to the soft tissues and to the vascularization, but it has been associated with postoperative shoulder pain and dysfunction. During the latest years a straight nail design was developed to minimize these problems.

**Methods:**

A total of 243 patients who had undergone surgery for antegrade intramedullary humeral nailing between January 2013 and July 2018 in A. Gemelli Trauma Center were divided into two groups according to the nail design used: straight nail group (S-group) and curvilinear nail group (C-group). Clinical data were collected using assessment forms (SF12-v2, Quick-DASH, ASES and Constant-Murley). Radiographic bone healing was assessed with RUST score at 30, 90 and 180 days after surgery.

**Results:**

The S-group was made up of 128 patients with a mean age of 59 ± 19 (range 18–97) and a mean follow-up of 46 ± 9 months. The C-group was made up of 115 patients with a mean age of 53 ± 16 (range 18–88) and a mean follow-up of 51 ± 8 months. The S-group had a mental component summary (MCS) score of 54.3 ± 7.7 and a physical component summary (PCS) score of 46 ± 10.2, the C-group had a MCS score of 50.9 ± 8.4 and a PCS score of 44.1 ± 7.4. Quick-DASH and ASES were respectively 18.8 ± 4.3 and 78.6 ± 8.2 in the S-group, 28.3 ± 11.6 and 72.1 ± 13.5 in the C-group with statistical significance. Constant-Murley score was 73.9 ± 9.1 in the S-group (76% of the contralateral healthy side) and 69.4 ± 10.4 in the C-group (73% of the contralateral healthy side). The radiographic union score in the S-group was 4.1 ± 0.3 at 30 days after surgery, 7 ± 0.8 at 90 days and 10 ± 1.2 at 180 days, while in the C-group it was 4.2 ± 0.4 at 30 days, 6.4 ± 0.7 at 90 days and 9 ± 0.9 at 180 days.

**Conclusion:**

Newer generation straight nails allow a faster bone healing and better functional outcome at mid-term follow up.

*Level of evidence* III.

## Introduction

Humerus fractures are common, accounting for 7–8% of all fractures in adults [1]. The incidence of humeral diaphysis fractures is 14 per 100,000 person a year, while the incidence of proximal humerus fractures is 63 per 100,000 person a year with patients over 60 involved in 70% of cases, representing the third most common fracture in the elderly after proximal femur and distal radius fractures [1–4]. Approximatively 80% of humeral fractures are non-displaced or minimally displaced and they can be treated conservatively with good functional results [5]. The remaining 20% are displaced and need surgery. Currently, many surgical options are available for the treatment of humeral fractures but the optimal management is still controversial and the treatment choice depends on the fracture pattern, on the surgeon’s preference and on the functional request of the patient [6, 7].

Antegrade intramedullary nail (IMN) fixation is a safe and effective technique: its main advantage is biological, preventing further damage to the soft tissues and preserving the vascularization of the fragments [8]. Moreover, it also provides a biomechanical advantage because the lever arm of the screws is lower than in plates thanks to its central position [9]. Nevertheless, it has been associated with a difficult indirect reduction of complex fractures and with the morbidity of the rotator cuff [10, 11]. Other complications such as varus malalignment and iatrogenic fracture of the greater tuberosity caused by a lateral entry point, and loss of fixation due to osteoporosis, have been described [12–14].

A curvilinear shape nail design was initially used for the treatment of these fractures. However, since this was associated with all the complications mentioned above, a straight intramedullary nail design was developed to minimize their incidence. To our knowledge there is a lack in the literature of studies comparing different intramedullary nail designs and their clinical outcomes.

The purpose of this study was to compare clinical and radiographic outcomes in patients who had undergone antegrade IMN fixation for proximal humerus and humeral diaphysis fractures using different nail designs.

## Methods

### Setting

A single center multi-surgeon retrospective study was performed, selecting all the patients who had undergone surgery for antegrade intramedullary humeral nailing between January 2013 and July 2018 in a high-level Trauma Center (Agostino Gemelli Hospital, Rome, Italy).

Inclusion criteria:

- Adult patients (over 18 years)
- Simple or complex fracture in the proximal or diaphyseal region of the humerus (11A2.1, 11A2.2, 11A2.3, 11A3, 11B1.1, 11B1.2, 12A1, 12A2, 12A3, 12B2, 12B3, 12C2, 12C3 according to AO classification) [15]

Exclusion criteria:

- Patients under the age of 18
- Patients suspected of pathological fractures or infections
- Patients with bilateral humeral fracture
- Evidence of neurological or vascular lesions
- Association of severe abdominal, thoracic or cranial trauma
- All patients subjected to other forms of treatment (surgical or not) before arrival in the hospital

The enrolled patients were divided into 2 groups according to the design of the IMN used: straight nail group (MultiLoc®, Depuy-Synthes, West Chester, PA, USA) and curved nail group (UHN® Depuy-Synthes, West Chester, PA, USA). The choice of nail type was based on the surgeon's preferences and personal experience.

Clinical data were collected using subjective quality of life assessment forms (SF12-v2) [16], quality of life related to specific disabilities assessment forms (Quick-DASH [17], ASES score [18]) and objective functional assessment forms (Constant-Murley score [19]). The follow up end points was set to 2 years of follow up.

**Fig. 1:**
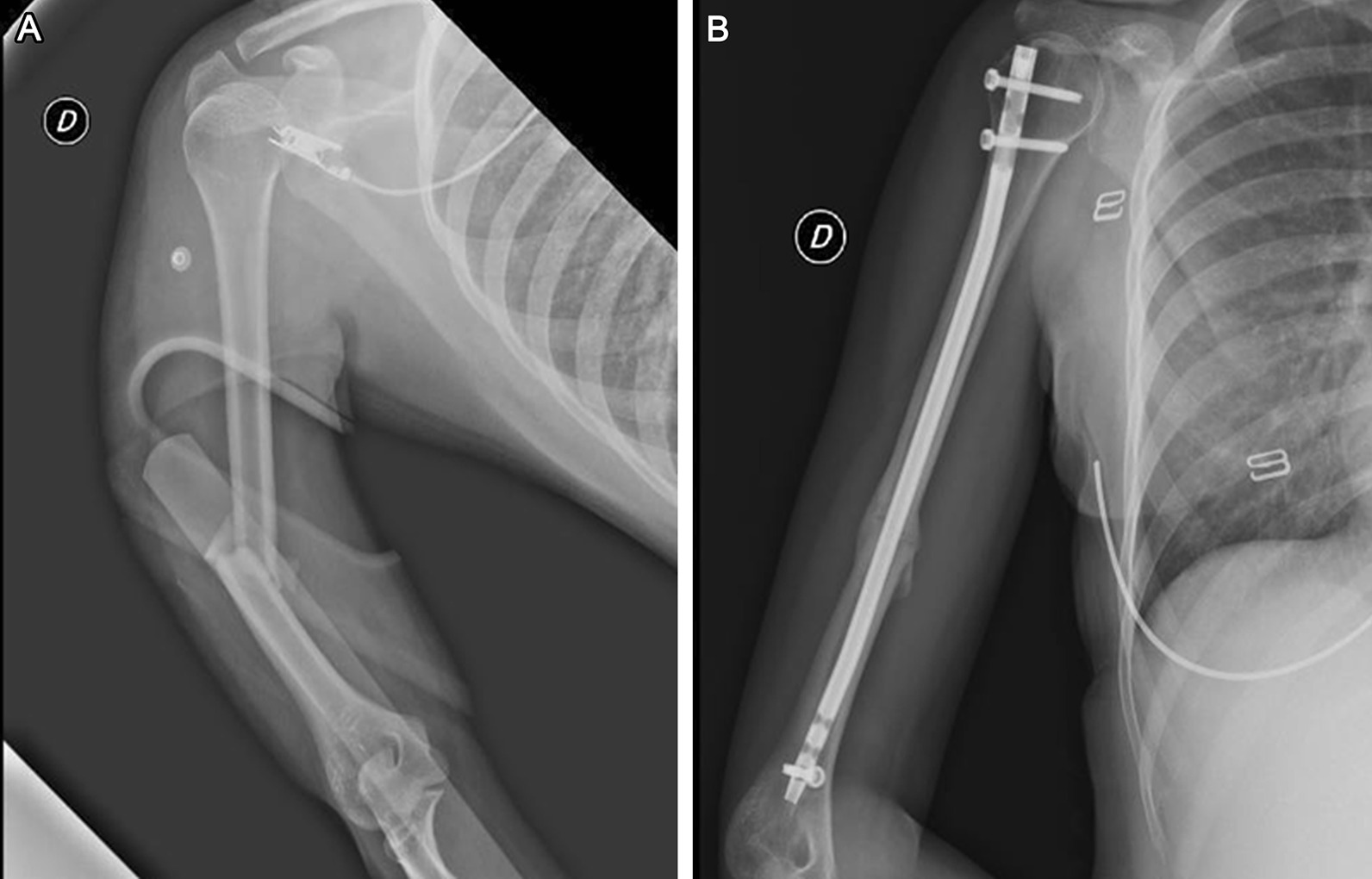
**A** Radiograph shows a fracture of humeral diaphyseal segment with a wedge fragment (AO cl. 12B2). **B** Good fracture healing at 180 days post-op after osteosynthesis with a curvilinear design intramedullary nail

**Fig. 2:**
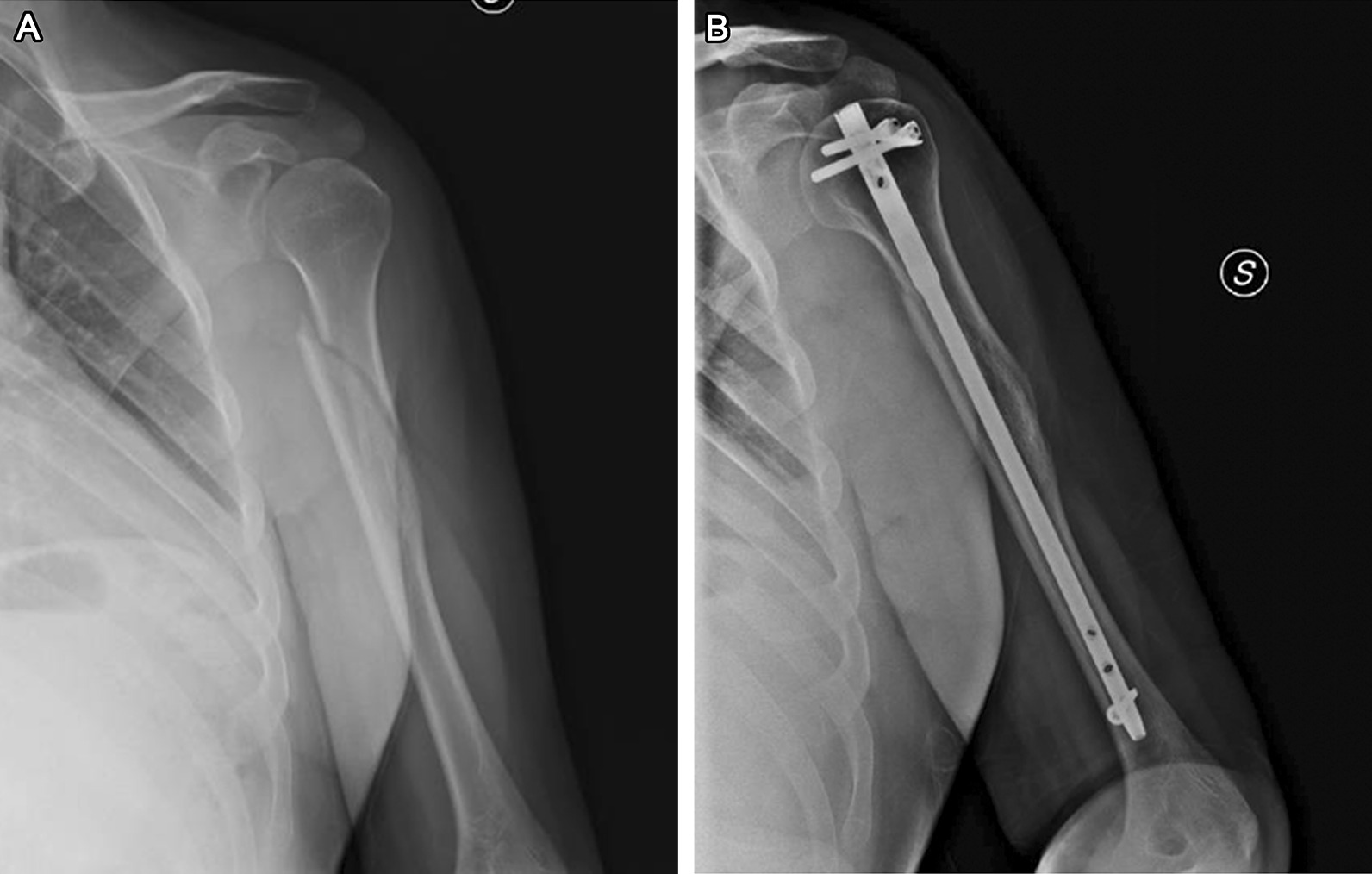
**A** Radiograph shows a spiral fracture of humeral diaphyseal segment (AO cl. 12A1). **B** Good fracture healing at 180 days post-op after osteosynthesis with a straight design intramedullary nail

X-ray examinations (AP and lateral view) were performed at 30, 90 and 180 days after surgery in all cases (Figs. 1, 2). To assess the bone healing the radiographic union score (RUST) as described by Whelan et al. was used [20]. This system assigns a score from 1 to 4 to each cortical visible in anteroposterior and lateral views, based on whether or not the callus is present and whether or not the fracture line is visible. This score goes from a minimum of 4 in which the fracture shows no sign of healing to a maximum of 12 in which the fracture is completely healed. Basic information such as age, sex, time elapsed since surgery, dominance was also included. The incidence of any complication was recorded.

### Surgical technique and post-operative treatment

General anesthesia, interscalene regional anesthesia, or a combination of both was performed. The patient was placed in beach chair position, with the image intensifier positioned posterior to the patient's back. Following standard preparation and draping, an anterolateral deltoid splitting approach was used in all cases. A small longitudinal incision of the rotator cuff was performed, parallel to the direction of the muscle fibers. After performing reduction (close or open, if necessary), under fluoroscopic guidance, the entry point for the nail was localized with a 2.0-mm guide wire (according to the nail design) and the nail was inserted. The length of the device was based on the anatomical position of the fracture identified during the preoperative radiographic examination and on the intraoperative assessments of the stability of the fracture. Once the fixation was completed, the rotator cuff was repaired with interrupted #2 absorbable side to side sutures. The wound was closed in layers.

The same rehabilitation protocol was used for the two groups, basically orienting the patient to remain with a simple sling on the operated side for a good analgesic condition. After two weeks from the surgery, the patient was encouraged to stimulate passive range of motion exercises, according to pain sensitivity. After four weeks from the surgery, activities of strengthening and elongation of external rotators and scapular stabilizers were initiated.

### Statistical analysis

Continuous variables were reported using means, standard deviations and ranges. Mean age of patients was rounded at the closest year. Outcome scores and their ranges were approximated at the first decimal. T-test was used to compare continuous variables, while *χ*^2^ test was used to compare categoric variables. The statistical significance was defined as *p* < 0.05. Statistical analysis was performed using SPSS (Armonk, NY: IBM Corp.).

## Results

### Participants

A total of 326 patients had undergone surgery in the time interval considered. 271 of them met the inclusion criteria and were eligible for the study. 5 patients died and 23 refused to participate or were impossible to reach. The remaining 243 patients were included in the study.

### Descriptive data

The straight nail group (S-group) included 128 patients with a mean follow-up of 46 ± 9 months (range 24–76). The curvilinear nail group (C-group) was made up of 115 patients with a follow-up of 51 ± 8 months (range 24–81). There were no significant differences in demographic characteristics between the S- and C- groups. Mean age in years of the S-group patients was 59 ± 19 (range 18–97) and in the C-group 53 ± 16 (range 18–88). The male to female ratio in each group was 1:1,2 in the S-group and 1:1 in the C-group. The most frequently affected side was the right side in both groups and this was the dominant side in 68% of the S-group and in 63% of the C-group (Table 1). The S-group was composed of 55 fractures of the proximal humerus and 73 fractures of the humeral shaft, while the C-group was composed of 39 fractures of the proximal humerus and 76 fractures of the humeral shaft (Table 2).

**Table 1:** Patients’ demographic

	Straight nail (*n* = 128)	Curvilinear nail (*n* = 115)
Age	59 ± 19 (range 18–97)	53 ± 16 (range 18–88)
Gender	57 M/71F	56 M/59F
Side	60L/68R	53L/62R
Dominant side	68%	63%
Follow up (months)	46 ± 9 (range 24–76)	51 ± 8 (range 24–81)

**Table 2:** Fracture classification

	Straight nail (*n* = 128)	Curvilinear nail (*n* = 115)
Proximal humerus	55	39
11-A2	15	9
11-A3	12	6
11-B1	16	14
11-B2	12	10
Humeral shaft	73	76
12-A1	18	23
12-A2	9	13
12-A3	13	8
12-B2	22	19
12-B3	1	3
12-C2	6	7
12-C3	4	3

### Functional outcome data

In almost all patients the return to a satisfactory quality of life was obtained: the S-group had a mental component summary (MCS) score of 54.3 ± 7.7 and a physical component summary (PCS) score of 46 ± 10.2, the C-group had a MCS score of 50.9 ± 8.4 and a PCS score of 44.1 ± 7.4, although not statistically significant. Quick-DASH and ASES were respectively 18.8 ± 4.3 and 78.6 ± 8.2 in the S-group, 28.3 ± 11.6 and 72.1 ± 13.5 in the C-group with statistical significance. Constant-Murley score was 73.9 ± 9.1 in the S-group (76% of the contralateral healthy side) and 69.4 ± 10.4 in the C-group (73% of the contralateral healthy side), but not statistically significant (Table 3).

**Table 3:** Functional scores

	Straight nail (*n* = 128)	Curvilinear nail (*n* = 115)	*p* value
SF-12 v2	MCS 54.3 ± 7.7	MCS 50.9 ± 8.4	0.23
	PCS 46 ± 10.2	PCS 44.1 ± 7.4	0.11
Quick-DASH	18.8 ± 4.3 (range 9–28)	28.3 ± 11.6 (range 4–43)	0.01*
ASES	78.6 ± 8.2 (range 58–88)	72.1 ± 13.5 (range 33–82)	0.005*
Constant-Murley	73.9 ± 9.1 (range 61–84)	69.4 ± 10.4 (range 59–80)	0.18
	[76% of contralateral]	[73% of contralateral]	

*Statistical significance (*p* < 0.05)

### Radiographic outcome data and complications

The radiographic union score in the S-group was 4.1 ± 0.3 30 days after surgery, 7 ± 0.8 90 days after surgery and 10 ± 1.2 180 days after surgery, while in the C-group it was 4.2 ± 0.4 30 days post-surgery, 6.4 ± 0.7 90 days post-surgery and 9 ± 0.9 180 days post-surgery. In 7 cases (4 in the S-group and 3 in the C-group) the fracture was unhealed after 180 days, 5 of them were successfully treated with extracorporeal shock wave therapy (ESWT) and/or pulsed electromagnetic fields (PEMF), 2 cases underwent revision surgery at 8 months with nail removal, bone graft and plate fixation. In both cases a complete radiographic bone healing was achieved at final follow up examination. No cases of post-operative infections have been reported.

(Table 4).

**Table 4:** Radiographic union score

	Straight nail (*n* = 128)	Curvilinear nail (*n* = 115)	*p* value
30 days	4.1 ± 0.3	4.2 ± 0.4	0.15
90 days	7 ± 0.8	6.4 ± 0.7	0.004*
180 days	10 ± 1,2	9 ± 0,9	0.001*
Complications	5	4	
Non-union	4	3	
Revision surgery	1	1	

*Statistical significance (*p* < 0.05)

## Discussion

The study showed better clinical and radiographic outcomes of straight design nails compared to curved design nails at mid-term follow up.

Humeral nails design have had many technical developments in the last decades and now its indication has extended to many fracture patterns [7, 21–24]. Chronic shoulder pain is a common complication after antegrade humeral nailing. Although the precise etiology has not been yet clearly defined and it probably is multifactorial (prominent hardware, shoulder impingement, stiffness, varus collapse, greater tuberosity to humeral head altered distance), it is believed to be mainly caused by the rotator cuff incision during nail insertion or damages in the critical insertion zone of the supraspinatus tendon [25]. The use of a straight design nail allows a more medial access to the humeral head, passing through the muscular fibers of the supraspinatus that have a higher biological healing capacity than the tendon. This could theoretically decrease the incidence of postoperative cuff tendinopathy [21]. Schwarz et al. compared the risk of iatrogenic tendon damage on a cadaveric model in 40 humeri showing that the lesions to the infraspinatus and long head of biceps tendons were different depending on the entry point [26]. A randomized clinical trial comparing straight and curvilinear design nails was published by Lopiz et al. in 2014 [27]. In a population of 54 patients with a mean follow-up of 14 months, they demonstrated that the Constant score was better in the group of patients treated with a straight design nail (61.2 ± 9.3 vs 51.4 ± 11.5), although not statistically significant. Moreover, patients in the straight design nail group complained much less of rotator cuff tendinopathy symptoms than patients in the curvilinear design nail group (34.6% vs 73%), with a statistic significance (*p* < 0.001). The straight design nail group had also a lower reoperation rate due to loss of fixation or to prominent hardware (11.5% vs 42%). A straight design nail has a biomechanical advantage: it allows a better alignment of the fracture and catches the densest part of the humeral head, theoretically improving the stability of the fixation and avoiding loss of reduction. Furthermore, it has been shown to preserve a greater bone stock [28]. Nolan et al. reported 94% incidence of healing using a straight nail for the treatment of proximal humerus fractures in 18 patients with a mean age of 71 years at 42 months of mean follow-up [29]. Gracitelli et al. showed in a literature review that in a total population of 1221 patients the treatment with intramedullary nails of 2 or 3-part humeral fractures is satisfactory, with curvilinear intramedullary nails having poorer results comparing to straight nails. Regarding four-fragments fractures, plate fixation has superior results [30]. In another recent study by Hao et al. 22 patients treated with a straight design nail had a mean Constant-Murley score of 75.5 and an ASES score of 81.7 at 12 months of mean follow-up. The average ROM was 144.3° in forward flexion, 141.3° in abduction, 58° in external rotation and 62° in internal rotation [31]. Hessmann et al. reported good results after antegrade fixation with a straight design nail at short term follow up (6 months) with a mean Constant-Murley score of 66.1, mean forward flexion of 134° and mean abduction of 125° and with a 100% of radiographic healing [23]. Moreover the recent work of Helfen et al. demonstrated good functional outcomes using straight design nails in 2-part fractures of the proximal humerus: both functional outcome and radiographic evaluation were satisfactory at 24 months of follow up, comparable to results achieved with the use of a locking plate fixation [32]. Kloub et al. also described satisfactory results of the use of a straight design nail even in 4-part fractures of the proximal humerus: in a cohort of 35 patients at 25,8 months of follow up a relative side related Constant-Murley score of 66.8% was obtained, with only 6 cases of avascular necrosis of the humeral head [24]. Muccioli et al. reported good clinical and radiographic outcomes in 40 patients treated with new generation straight design nails (34 Aequalis Tornier Wright and 6 Multiloc Depuy Synthes) with a mean Constant-Murley score of 70 ± 17 at 8 months of follow up. They also performed an ultrasound examination that found 5 supraspinatus tendon tears (12,5%) without functional significance and 8 cases of tenosynovitis of long head of biceps. A rotator cuff integrity was found in the remaining shoulders [33]. Finally, the complication rate of the present study was 3.7%, that is consistent with the complication rate reported in the recent literature [34]. No case of infection was reported, maybe thanks to the small surgical approach used and to the closed reduction achieved in most cases.

The present study has certain obvious limitations. The first limitation is due to its retrospective design. Secondly, the variability of the population, the heterogeneity of fractures, and the number of surgeons included. Another limitation is the absence of information regarding rotator cuff pathology previous to the fracture. Therefore, further studies with higher levels of evidence are needed to address these questions thoroughly.

## Conclusion

Functional recovery after antegrade intramedullary nailing for proximal and shaft humerus fractures is satisfactory at mid-term follow up. Radiographic bone healing is obtained within 180 days in the majority of cases. The use of newer generation straight nails allows a quicker complete bone healing and minimizes pain and dysfunction, providing better functional outcome.

## Data Availability

The datasets used and/or analyzed during the current study are available from the corresponding author on reasonable request.

## Abbreviations

MCS: Mental component summary
PCS: Physical component summary
S-group: Straight nail group
C-group: Curvilinear nail group
IMN: Antegrade intramedullary nail
